# The Expression and Prognostic Roles of MCMs in Pancreatic Cancer

**DOI:** 10.1371/journal.pone.0164150

**Published:** 2016-10-03

**Authors:** Yun-Peng Peng, Yi Zhu, Ling-Di Yin, Jing-Jing Zhang, Song Guo, Yue Fu, Yi Miao, Ji-Shu Wei

**Affiliations:** 1 Pancreas Institute of Nanjing Medical University, Nanjing, 210029, People’s Republic of China; 2 Pancreas Center, The First Affiliated Hospital of Nanjing Medical University, Nanjing, 210029, People’s Republic of China; 3 Department of General Surgery, The first Affiliated Hospital of Nanjing Medical University, Nanjing, 210029, People’s Republic of China; Saint George's University, UNITED KINGDOM

## Abstract

**Objectives:**

Minichromosome maintenance (MCM) proteins play important roles in DNA replication by interacting with other factors which participate in the regulation of DNA synthesis. Abnormal over-expression of MCMs was observed in numerous malignancies, such as colorectal cancer. However, the expression of MCMs in pancreatic cancer (PC) was less investigated so far. This study was designed to analyze the expression and prognostic roles of MCM1-10 in PC based on the data provided by The Cancer Genome Atlas (TCGA).

**Methods:**

Pearson χ^2^ test was applied to evaluate the association of MCMs expression with clinicopathologic indicators, and biomarkers for tumor biological behaviors. Kaplan-Meier plots and log-rank tests were used to assess survival analysis, and univariate and multivariate Cox proportional hazard regression models were used to recognize independent prognostic factors.

**Results:**

MCM1-10 were generally expressed in PC samples. The levels of some molecules were markedly correlated with that of biomarkers for S phase, proliferation, gemcitabine resistance. And part of these molecules over-expression was significantly associated with indicators of disease progression, such as depth of tumor invasion and lymph node metastasis. Furthermore, MCM2, 4, 6, 8, and 10 over-expression was remarkably associated with shorter disease free survival time, and MCM2, 4,8, and 10 over-expression was associated with shorter overall survival time. Further multivariate analysis suggested that MCM8 was an independent prognostic factor for PC.

**Conclusion:**

MCMs abnormal over-expression was significantly associated with PC progression and prognosis. These molecules could be regarded as prognostic and therapeutic biomarkers for PC. The roles of MCMs may be vitally important and the underlying mechanisms need to be furtherinvestigated.

## Introduction

Pancreatic cancer is a digestive malignancy with extremely aggressive behavior. It is the fifth leading cause of cancer-related deathsworldwide, and the five-year survival rate of such cancer is approximately8% on the basis of latest data provided by Siegel RL, et al [1]. Chemotherapy and radiotherapy are not effective for PC due to several reasons, such as complex genetic mutations, hypoxic tolerance, and excessive fibrosis [2]. At present, the only available treatment for PC is radical operation; however, the rate of surgical resection is less than 20% because of the absence of obvious symptoms at the early stage [3].Therefore, to find novel therapeutic strategies based on molecular biomarkers of PC is extremely urgent. Recently, an increasing number of molecular contribute to the transformation and progression of PC were identified, some of these molecular was regarded as prognostic and/or therapeutic markers, such as MUC4, LSD1, and FHL2 [4–6].

MCM family is composed of ten proteins which primarily promote the process of DNA replication of eukaryotes [7]. MCM1 is an important member of MADS box transcription factor family, this protein affects the process of cell cycle, apoptosis, growth, and differentiationthrough regulating many gene activation [8]. The MCM2-7 heterohexamer complex was first detected in the yeast *Saccharomyces cerevisiae*[9], and the functions of this complex were extensively studied nowdays.MCM2-7interact with each other to form a functional DNA helicases which trigger the initial step of DNA synthesis [10, 11]. MCM8 and MCM9 were generally considered as additional members of MCM2-7 family [7]. Like MCM2-7 complex, MCM8 and MCM9 were also crucial components of the pre-replication complex. MCM8 and MCM9 were also involved in drivingthe initiation of Sphase [12, 13]. MCM10 is another necessary molecule for initializing the DNA synthesis due to its interaction with MCM2-7 complex[14, 15]. Furthermore, it has been reported that some members ofMCMs were abnormally up-regulated in various malignancies, and over-expression of them could promote the progression of malignant cells and predict the survival times of suffered patients [16–18]. However, the roles of MCMs in PC was absolutely unknown.

Here we will assess the expression of MCMs in PC according to the data provided by TCGA, and analyze the association between MCMs expression and the progression, prognosis of PC.

## Methods and Materials

### Clinicopathologic Features and MCMs Expression

Clinicopathologic features and MCMs expression (level 3 data, log2(RSEM+1) transformed) for pancreatic cancer patients were downloaded from TCGA data portal (http://cancergenome.nih.gov/). The National Cancer Institute (NCI) and National Human Genome Research Institute (NHGRI) work with physicians who collect tissue for TCGA to gain approval with local Institutional Review Boards (IRBs). An IRB is a group of scientists, doctors, clergy and consumers who review and approve the research proposal for every research project that involves human subjects. 165 patients were finally included in our study, others were excluded due to the lack of critical information (such as overall survival time, age, gender, et al). Main clinicopathologic features for PC patients were shown in **Table 1**. All patients were divided into low and high expression group according to the median value of each gene expression. Gene was defined as high expression if this gene expression was more than or equal to median value, otherwise it was defined as low expression.

**Table 1:** Clinicopathologic features of the patients with PC.

		Pancreatic Cancer Patients (n = 165)
Age	Median age	65
	Range	35–88
Gender	Male	90(54.55%)
	Female	75(45.45%)
TMN stage	0/I/IIA	0(0.00%)/20(12.12%)/25(15.15%)
	IIB/III/IV	113(68.48%)/4(2.42%)/3(1.82%)

### Statistical Analysis

The different expression groups (high *vs*. low) was defined by the median value of MCMs expression. The association of MCMs expression with clinicopathologic indicators was accessed by Pearson χ^2^ test. The correction between MCMs expression and overall survival time was evaluated by Kaplan-Meier plots and log-rank tests, as well as disease free time. Independent prognostic factors were recognized byunivariate and multivariate Cox proportional hazard regression models were used to recognize. All statistical analyses were performed by SPSS 20.0 software. Differences between groups were considered significant at *P*<0.05.

## Results

### MCMs Expression and Association with Biomarkers for Tumor Biological Behaviors

**Fig 1:**
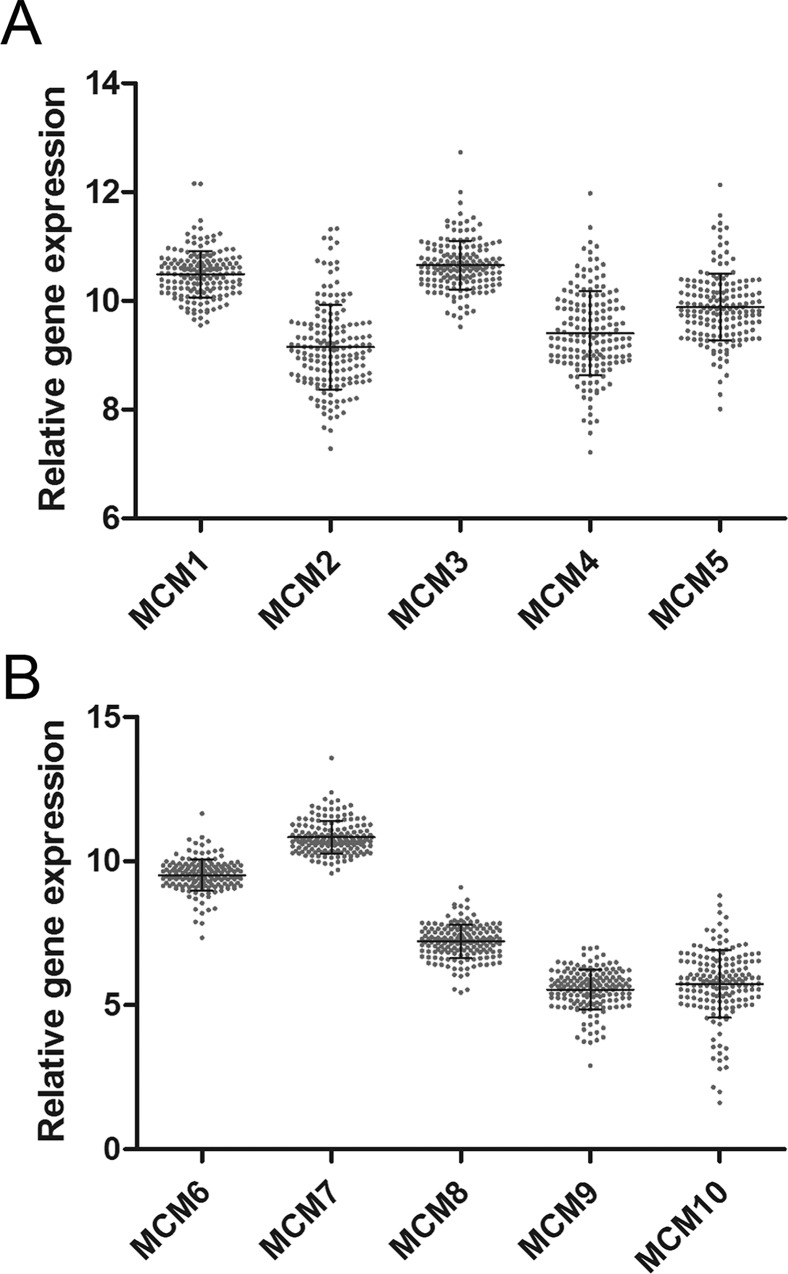
The relative expression of MCM1-10 in 165 PC samples. (A) The relative expression of MCM1-5 in 165 PC samples. (B) The relative expression of MCM6-10 in 165 PC samples.

As shown in Fig 1, all MCMs were generally determined in PC samples.

As MCMs were reported to participate in the synthesis of DNA, we firstly analyzed the association between MCMs and markers for S phase and proliferation. MCM2-7 complex, MCM8, and MCM10 expression was positively associated with CDK1 and CCNB1 expression which worked as critical proteins in S phase. Almost all assessed molecules were positively corrected with the proliferative biomarker Ki-67 in addition to MCM1. We further assessed the functions of MCMs in cell invasion, migration, and EMT. A few molecules were weakly or moderately associated with MMP2, MMP7, and MMP9; little correlation between MCMs expression and EMT biomarkers expression was observed. Furthermore, we evaluated the roles of MCMs in gemcitabine resistance, and analytic data suggested that most of these molecules over-expression was associated with gemcitabine resistance biomarker RRM1 in different degrees. All results in this aspect were shown in **Table 2**. These results demonstrated that higher MCMs levels might be responsible for PC cell proliferation and gemcitabine resistance.

**Table 2:** Association between MCMs and biomarkers for tumor biological behaviors.

		CDK1	CCNB1	Ki67	RRM1	hENT	MMP2	MMP7	MMP9	ECAD	VIM
MCM1	r	-0.162	-0.276	-0.104	-0.088	0.211	0.357	0.062	0.112	-0.291	0.36
	*P*	0.038	0.000	0.184	0.264	0.006	0.000	0.428	0.152	0.000	0.000
MCM2	r	0.639	0.675	0.606	0.666	-0.126	0.114	0.036	0.222	0.026	0.015
	*P*	0.000	0.000	0.000	0.000	0.106	0.145	0.643	0.004	0.738	0.850
MCM3	r	0.460	0.439	0.485	0.408	0.114	0.009	0.102	0.129	-0.055	0.028
	*P*	0.000	0.000	0.000	0.000	0.146	0.912	0.194	0.097	0.480	0.720
MCM4	r	0.660	0.681	0.678	0.667	-0.086	0.130	0.124	0.163	0.184	-0.123
	*P*	0.000	0.000	0.000	0.000	0.274	0.097	0.112	0.037	0.018	0.115
MCM5	r	0.419	0.382	0.409	0.341	0.024	0.106	0.082	0.401	-0.197	0.200
	*P*	0.000	0.000	0.000	0.000	0.760	0.175	0.298	0.000	0.011	0.010
MCM6	r	0.613	0.544	0.609	0.564	-0.029	0.423	0.235	0.330	-0.129	0.271
	*P*	0.000	0.000	0.000	0.000	0.707	0.000	0.002	0.000	0.097	0.000
MCM7	r	0.453	0.574	0.390	0.429	-0.124	-0.148	-0.141	0.060	-0.006	-0.181
	*P*	0.000	0.000	0.000	0.000	0.112	0.057	0.071	0.446	0.942	0.020
MCM8	r	0.509	0.469	0.542	0.472	-0.119	0.035	-0.053	0.179	-0.008	-0.149
	*P*	0.000	0.000	0.000	0.000	0.128	0.660	0.501	0.022	0.915	0.056
MCM9	r	0.067	-0.152	0.190	-0.081	0.054	0.135	0.287	0.113	0.044	-0.066
	*P*	0.394	0.052	0.015	0.302	0.489	0.083	0.000	0.149	0.579	0.406
MCM10	r	0.889	0.731	0.857	0.569	-0.038	0.230	0.339	0.321	0.172	-0.013
	*P*	0.000	0.000	0.000	0.000	0.625	0.003	0.000	0.000	0.027	0.868

### Association between MCMs Expression and Clinicopathologic Variables

Analytic results suggested that MCM4 over-expression was detected in patients with aggressive T stage, and MCM9 was over-expressed in patients with lymph node metastasis (**Table 3**). Furthermore, no other association between these genes expression and clinicopathologic features were observed (**Table 3**). Results in this aspect suggested that MCM4 and MCM9 could be considered as biomarkers for PC progression.

**Table 3:** Association between MCMs expression and clinicopathologic variables.

		Histology stage		N stage		T stage		TNM stage	
		Well or moderate	Poor	Absent	Present	T1 or T2	T3 or T4	I-IIA	IIB-IV
MCM1	Low	54	29	25	58	16	67	23	60
	High	62	20	23	59	11	71	22	60
	*P*	0.138		0.770		0.309		0.899	
MCM2	Low	64	19	23	60	16	67	23	60
	High	52	30	25	57	11	71	22	60
	*P*	0.054		0.695		0.309		0.899	
MCM3	Low	61	22	23	60	17	66	23	60
	High	55	27	25	57	10	72	22	60
	*P*	0.367		0.695		0.150		0.899	
MCM4	Low	63	20	25	58	19	64	24	59
	High	53	29	23	59	8	74	21	61
	*P*	0.113		0.770		**0.023**		0.634	
MCM5	Low	62	21	27	56	15	68	25	58
	High	54	28	21	61	12	70	20	62
	*P*	0.214		0.328		0.551		0.409	
MCM6	Low	59	24	27	56	16	67	26	57
	High	57	25	21	61	11	71	19	63
	*P*	0.825		0.328		0.309		0.240	
MCM7	Low	63	20	22	61	17	66	22	61
	High	53	29	26	56	10	72	23	59
	*P*	0.113		0.462		0.150		0.824	
MCM8	Low	63	20	25	58	15	68	23	60
	High	53	29	23	59	12	70	22	60
	*P*	0.113		0.770		0.551		0.899	
MCM9	Low	64	19	30	53	16	67	27	56
	High	52	30	18	64	11	71	18	64
	*P*	0.054		**0.045**		0.309		0.127	
MCM10	Low	61	22	22	60	17	66	22	61
	High	55	27	25	57	10	72	23	59
	*P*	0.367		0.695		0.150		0.824	

### Survival Outcomes and Multivariate Analysis

**Fig 2:**
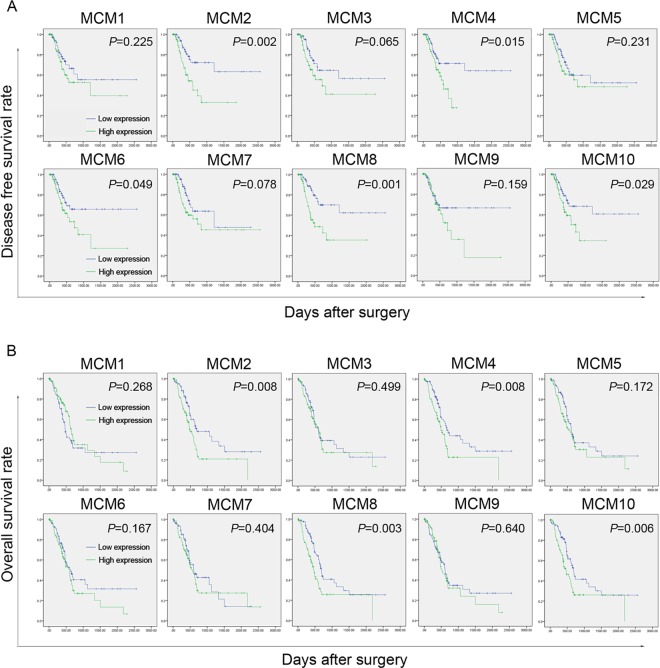
Survival analysis of PC patients correlated with MCM1-10 expression. (A) The association between MCM1-10 expression and disease free survival time. The higher expression of MCM2, 4, 6, 8, and 10 was significantly associated with shorter disease free survival time. (B) The correlation between MCM1-10 expression and overall survival time. The higher levels of MCM2, 4, 8, and 10 were markedly corrected with poorer outcome.

Firstly, the influence of MCMs on disease free survival time was evaluated. A total of 140 patients with disease free survival time related data were enrolled in this section. Analytic results suggested that MCM2, 4, 6, 8, and 10 expression was significantly associated with disease free survival time (**Fig 2A**). Specifically patients with lower MCM2, 4, 6, 8, 10 levels had longer disease free survival time.And then, the association between MCMs and overall survival time was also assessed. Similar to disease free survival time, lower MCM2, 4, 8, 10 expression was markedly correlated with better overall survival (**Fig 2B**). Other molecules did not show any correlation with overall survival. Finally, independent prognostic factors was investigated by using Cox proportional hazard regression models. Results of univariate analysis demonstrated that not only some MCM proteins but also some clinicopathologic indicators corrected with overall survival, including T stage, N stage, and TNM stage (**Table 4**). Multivariate analytic results suggested that MCM8 could independently predict the overall survival of PC (**Table 4**).

**Table 4:** Survival outcomes.

	Univariable analysis			Multivariable analysis		
	HR	95% CI	*P*	HR	95% CI	*P*
MCM1	0.784	0.509–1.207	0.270			
MCM2	1.786	1.154–2.734	0.009	1.322	0.700–2.498	0.389
MCM3	1.16	0.754–1.783	0.500			
MCM4	1.805	1.159–2.809	0.009	1.240	0.684–2.248	0.478
MCM5	1.349	0.876–2.076	0.174	1.126	0.687–1.845	0.639
MCM6	1.353	0.880–2.079	0.169	0.565	0.309–1.035	0.065
MCM7	1.201	0.780–1.850	0.405			
MCM8	1.895	1.227–2.928	0.004	1.707	1.047–2.863	0.032
MCM9	1.108	0.720–1.707	0.640			
MCM10	1.818	1.176–2.811	0.007	1.588	0.881–2.863	0.124
Age	1.456	0.942–2.250	0.091	1.448	0.917–2.288	0.112
Gender	0.934	0.608–1.434	0.755			
HS	1.556	0.990–2.445	0.055	1.172	0.725–1.895	0.517
N stage	2.001	1.193–3.358	0.009	1.169	0.358–2.816	0.796
T stage	2.396	1.186–4.838	0.015	1.383	0.621–3.080	0.427
TMN stage	2.323	1.333–4.046	0.003	2.052	0.557–7.554	0.280

CI, confidence interval; HR, hazard ratio; HS, Histology stage.

## Discussion

PC is a digestive malignancy with extremely high mortality and less therapeutic options. At present the most urgent work for this disease is to investigate novel and efficient biomarkers for prognosis and therapy [19]. In this study, we explored the roles of MCMs in PC for the first time. MCMs abnormal over-expression was significantly associated with PC progression, aggressive PC cell behaviors, poorer disease free survival, and poorer overall survival.

It has been reported thatMCM2-7 complex could work together as DNA helicases to promote the initial stage of DNA replication through participating the formation of pre-replication complex [20–22]. Once binding with some crucial factors in cell cycle (such as Cdc6, Cdt1, and Dbf4/Cdc7), MCM2-7 complex was activated to further enhance DNA synthesis by triggering DNA unwind [23–25]. In other words, MCM2-7 complex could promote cell cycle from S phase to G2/M phase, and finally enhance cell proliferation. Our analytic results also revealed MCM2-7 complex was very important in S phase. The expression of all members of MCM2-7 complex was positively associated with that of S phase biomarkers (CDK1 and CCNB1), as well as cell proliferation biomarker (Ki-67). However, there was no connection between MCM2-7 complex and PC cell migration, invasion, and EMT. Furthermore, MCM2-7 complex members were abnormally up-regulated in various cancers, such as gastric cancer and colon cancer. And they could be regarded as indicators for certain cancer progression and prognosis [26, 27].Our results also suggested that some members of MCM2-7 complex could predict PC progression (MCM4 for T stage). In accordance with the data provided by several past studies in other malignancies, PC patients with higher levels of MCM2, MCM4 and MCM 6 had poorer outcomes (MCM2, 4, and 6 for disease free survival time and MCM2 and 2 for overall survival time). However, none of them was an independent predictor of worse outcome.

There were other four members belong to MCM family, including MCM1, 8, 9, and 10. All of these four molecules were identified as critical factors in the regulation of cell cycle through different mechanisms. MCM1 could activate many immediate-early genes to further perform its function by interacting with serum response element [28, 29].As reported, the interaction between MCM8and CDC6 couldeffectively accelerate pre-replication complex assembly [30], as well as MCM9 and Cdt1 interaction [31]. It has also been reported that MCM8 and MCM9 could form a complex to facilitate homologous recombination which was mediated by RAD51 recruitment at DNA damage sites [32]. MCM10 was reported to promote chromosome replication by enhancing the assembly of the Cdc45-Mcm2-7-GINS complex [33]. And MCM10-RECQ4 interaction was necessary for this process [34]. All reported results above revealed that these four MCMs could also promote cell proliferation by influencing cell cycle. Our analytic data were similar to the report of literatures that MCM8-10 were positively correlated with biomarkers for S phase and cell proliferation in different degrees. Furthermore, many articles focused on these MCMs in malignancies demonstrated that their over-expression was significantly associated with clinicopathologic features for disease progression [17, 35, 36]. However, we just found that MCM9 over-expression was markedly associated lymph node metastasis of PC. Moreover, the prognostic values of MCM8 and MCM10 in PC were similar to MCM2 and MCM4, and MCM8 could be regarded as an independent prognostic factor for PC patients.

Furthermore, latest studies revealed that suppression of the MCMs could sensitize cancer cells to chemotherapeutic agents by inhibiting replicative fork progression, for example, gemcitabine and 5-Fluorouracil [37]. Therefore, we assessed the association between MCMs and gemcitabine resistance biomarkers. Meaningly, MCM2-7 complex expression was significantly associated with gemcitabine resistance biomarker RRM1, especially MCM2 and MCM4 which has high association with RRM1. And then, MCM7 and MCM9 was also moderately associated with RRM1. According to these results, we proposed a hypothesis that gemcitabine resistance might be improved after some MCMs down-regulation.

## Conclusion

In conclusion, abnormally up-regulated MCMs in PC were significantly associated with cancer cell proliferation, gemcitabine resistance, disease progression, and poorer outcomes. These results suggested that MCMs were potential biomarkers for PC progression and prognosis, and MCMs could also be considered as targets to improve the efficiency of gemcitabine-based therapy.

## Supporting Information

S1 File The primary data of MCM1-10 expression. (XLSX)

S2 File The survival and clinicopathologic information of PC patients enrolled in this study. (XLSX)

S3 File The primary data of some cancer biomarkers expression. (XLSX)
